# Supplementary material for: Portable, bedside, low-field magnetic resonance imaging for evaluation of intracerebral hemorrhage
Source: Nat Commun. 2021 Aug 25;12:5119. doi: 10.1038/s41467-021-25441-6 (PMC8387402; doi:10.1038/s41467-021-25441-6)
Supplement: Supplementary file 1 — Supplementary Information [file 41467_2021_25441_MOESM1_ESM.pdf]

Supplementary Information for

Portable, Bedside, Low-Field Magnetic Resonance Imaging for Evaluation of Intracerebral Hemorrhage

Mercy H. Mazurek[1*], Bradley A. Cahn[1*], Matthew M. Yuen[1], Anjali M. Prabhat[1], Isha R. Chavva[1], Jill T. Shah[1], Anna L. Crawford,[1] E. Brian Welch[2], Jonathan Rothberg[2], Laura Sacolick[2], Michael Poole[2], Charles Wira[3], Charles C. Matouk[4], Adrienne Ward[5], Nona Timario[5], Audrey Leasure[1], Rachel Beekman[1], Teng J. Peng[1], Jens Witsch[1], Joseph P. Antonios[4], Guido J. Falcone[1], Kevin T. Gobeske[1], Nils Petersen[1], Joseph Schindler[1], Lauren Sansing[1], Emily J. Gilmore[1], David Y. Hwang[1], Jennifer A. Kim[1], Ajay Malhotra[6], Gordon Sze[6], Matthew S. Rosen[7], W. Taylor Kimberly[8†], Kevin N. Sheth[1†]

Affiliations:
1. Department of Neurology, Yale School of Medicine, New Haven, CT
2. Hyperfine Research, Inc.
3. Department of Emergency Medicine, Yale School of Medicine, New Haven, CT
4. Department of Neurosurgery, Yale School of Medicine, New Haven, CT
5. Neuroscience Intensive Care Unit, Yale New Haven Hospital, New Haven, CT
6. Department of Radiology, Yale University School of Medicine, New Haven, CT, USA\
7. Athinoula A. Martinos Center for Biomedical Imaging, Massachusetts General Hospital, Charlestown, MA, USA
8. Department of Neurology, Division of Neurocritical Care, Massachusetts General Hospital, Boston, MA, USA

* These authors contributed equally to this work
† Co-corresponding authors

Correspondence:

Kevin N. Sheth, MD
15 York Street
LLCI Room 1003C
P.O. Box 208018
New Haven, CT 06520, USA
Phone: 203-737-8051
Email: kevin.sheth@yale.edu

W. Taylor Kimberly, MD, PhD
Massachusetts General Hospital
Lunder 644
55 Fruit Street
Boston, MA 02114
Phone: 857-238-5644
Email: wtkimberly@mgh.harvard.edu

Table S1. Lesion Localization on Portable MRI Compared to Conventional Imaging. To localize lesions between pMRI and conventional (CT or 1.5/3T MRI) examinations, the Euclidean distances between the centroids of the manually segmented hemorrhages and the centroids of each point ROI at the aforementioned anatomical locations were compared across modalities using Pearson correlations and Bland-Altman analyses.

Anatomical Location	pMRI Sequence	Pearson Correlation (95% CI)	*P* Value	Bland-Altman Bias (LOA)
Optic Chiasm	T2W	0.937 (0.879 to 0.9682	<2.20e-16	-2.260 (-16.6 to 12.1)
Optic Chiasm	FLAIR	0.913 (0.837 to 0.954)	1.46e-15	-1.742 (-18.7 to 15.2)
Septum Pellucidum	T2W	0.874 (0.763 to 0.935)	6.88e-12	-3.294 (-23.1 to 16.5)
Septum Pellucidum	FLAIR	0.954 (0.912 to 0.976)	<2.20e-16	0.2676 (-9.95 to 10.5)
Right Edge	T2W	0.866 (0.749 to 0.931)	1.80e-11	1.066 (-23.5 to 25.7)
Right Edge	FLAIR	0.895 (0.805 to 0.944)	3.67e-14	1.195 (-21.3 to 23.7)
Left Edge	T2W	0.964 (0.929 to 0.982)	<2.20e-16	-0.3898 (-14.3 to 13.6)
Left Edge	FLAIR	0.969 (0.940 to 0.984)	<2.20e-16	-0.2708 (-13.4 to 12.9)

Abbreviations: pMRI, portable magnetic resonance imaging; T2W, T2-weighted; FLAIR, fluid-attenuated inversion recovery; LOA, limits of agreement.

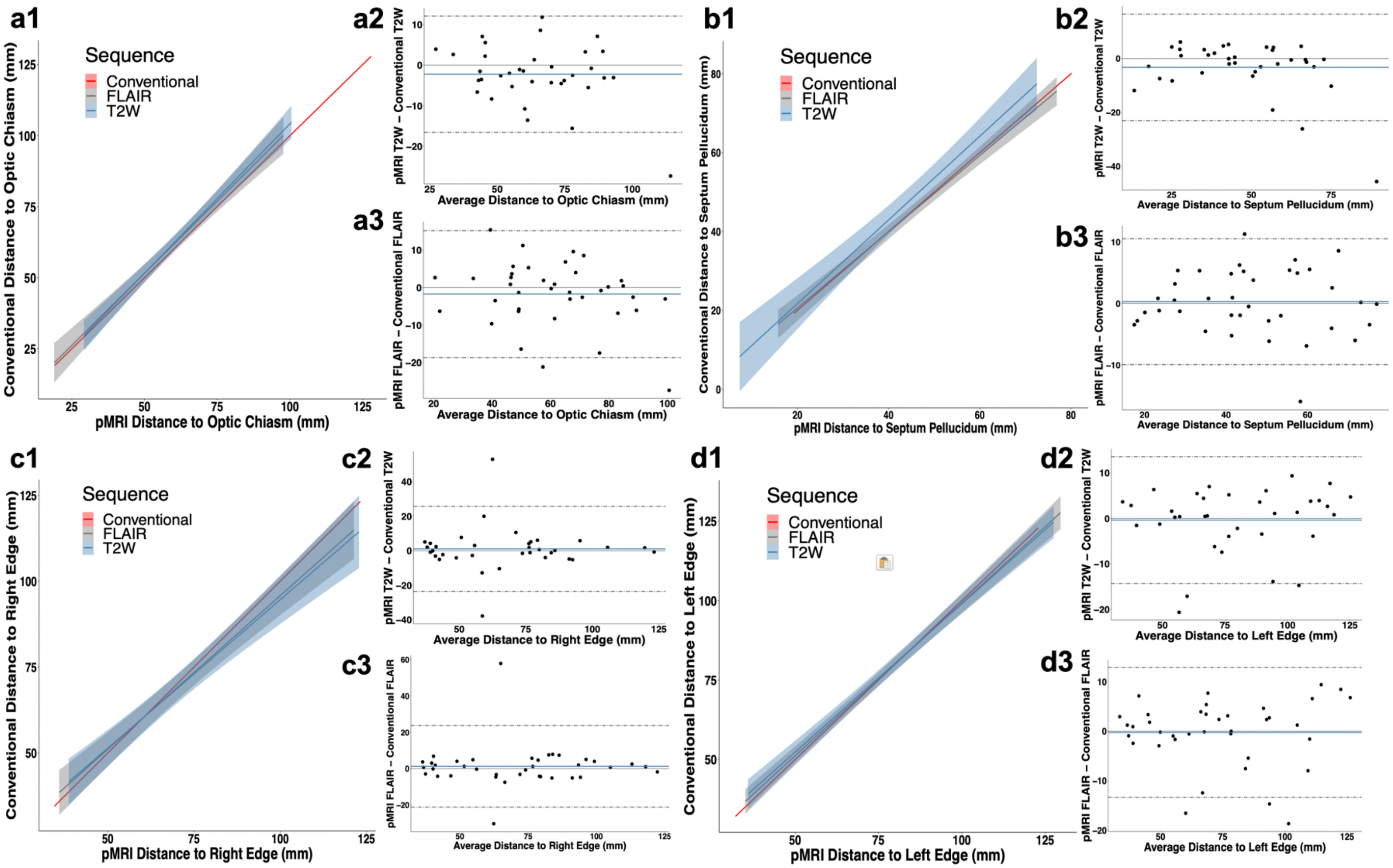

Figure S1. Lesion Localization on Portable MRI (pMRI) Compared to Conventional Imaging. To localize lesions across modalities, the Euclidean distances were computed between the centroids of the manually segmented hematomas and the centroids of point regions of interest (ROIs) at four anatomical locations. Euclidean distances on pMRI were correlated with those on conventional imaging for distances between the hematoma and the (a1-3) optic chiasm, (b1-3) septum pellucidum, (c1-3) anatomical right edge of the central sulcus at the topmost level of the lateral ventricles, and (d1-3) anatomical left edge of the central sulcus. Pearson correlations (a1, b1, c1, d1) and Bland-Altman (a2-3, b2-3, c2-3, d2-3) analyses are reported with confidence intervals. Line of identity is shown in red (a1-d1). 95% confidence intervals are represented by bands (a1, b1, c1, d1) and dashed gray lines (a2-3, b2-3, c2-3, d2-3).
