# Supplementary material for: Portable, bedside, low-field magnetic resonance imaging for evaluation of intracerebral hemorrhage
Source: Nat Commun. 2021 Aug 25;12:5119. doi: 10.1038/s41467-021-25441-6 (PMC8387402; doi:10.1038/s41467-021-25441-6)
Supplement: Supplementary file 3 — Reporting Summary [file 41467_2021_25441_MOESM3_ESM.pdf]

nature research

Corresponding author(s): Kevin N. Sheth

Last updated by author(s): Jul 22, 2021

Reporting Summary

Nature Research wishes to improve the reproducibility of the work that we publish. This form provides structure for consistency and transparency in reporting. For further information on Nature Research policies, see our Editorial Policies and the Editorial Policy Checklist.

Statistics

For all statistical analyses, confirm that the following items are present in the figure legend, table legend, main text, or Methods section.

n/a	Confirmed	
☐	☒	The exact sample size (*n*) for each experimental group/condition, given as a discrete number and unit of measurement
☐	☒	A statement on whether measurements were taken from distinct samples or whether the same sample was measured repeatedly
☐	☒	The statistical test(s) used AND whether they are one- or two-sided<br>*Only common tests should be described solely by name; describe more complex techniques in the Methods section.*
☐	☒	A description of all covariates tested
☒	☐	A description of any assumptions or corrections, such as tests of normality and adjustment for multiple comparisons
☐	☒	A full description of the statistical parameters including central tendency (e.g. means) or other basic estimates (e.g. regression coefficient) AND variation (e.g. standard deviation) or associated estimates of uncertainty (e.g. confidence intervals)
☐	☒	For null hypothesis testing, the test statistic (e.g. F, t, r) with confidence intervals, effect sizes, degrees of freedom and P value noted<br>*Give P values as exact values whenever suitable.*
☒	☐	For Bayesian analysis, information on the choice of priors and Markov chain Monte Carlo settings
☒	☐	For hierarchical and complex designs, identification of the appropriate level for tests and full reporting of outcomes
☐	☒	Estimates of effect sizes (e.g. Cohen's *d*, Pearson's *r*), indicating how they were calculated

Our web collection on statistics for biologists contains articles on many of the points above.

Software and code

Policy information about availability of computer code

Data collection	The pMRI underwent multiple software updates over the course of the study, including: software versions RC3, RC4, RC5, RC6, RC7, RC8.
Data analysis	Horos (v.3.3.5), RadiAnt (v.2020.2.3), RStudio (v.3.6.1), AFNI (v. 21.1.02)

For manuscripts utilizing custom algorithms or software that are central to the research but not yet described in published literature, software must be made available to editors and reviewers. We strongly encourage code deposition in a community repository (e.g. GitHub). See the Nature Research guidelines for submitting code & software for further information.

Data

Policy information about availability of data

All manuscripts must include a data availability statement. This statement should provide the following information, where applicable:

- Accession codes, unique identifiers, or web links for publicly available datasets
- A list of figures that have associated raw data
- A description of any restrictions on data availability

Source data are provided with this paper as a source data file. Raw data associated with Tables 1, 2, and 3 and Figures 4 and 5 are included in the source data file. While a public portable MRI neuroimaging repository is not established yet, there are ongoing efforts to make the neuroimaging data publicly available. In the interim, the neuroimaging studies analysed during the current study are available from the corresponding author upon request.

Field-specific reporting

Please select the one below that is the best fit for your research. If you are not sure, read the appropriate sections before making your selection.

☒ Life sciences ☐ Behavioural & social sciences ☐ Ecological, evolutionary & environmental sciences

For a reference copy of the document with all sections, see nature.com/documents/nr-reporting-summary-flat.pdf

Life sciences study design

All studies must disclose on these points even when the disclosure is negative.

Sample size	No statistical methods were used to compute a predetermined sample size. The sample size was selected based on a convenience sample including the number of subjects who received a portable MRI brain examination from July 2018 to November 2020 at Yale New Haven Hospital. Subjects who met the following inclusion criteria were included in the convenience sample: (1) confirmed diagnosis of intracerebral hemorrhage, ischemic stroke or no intracranial abnormality on available standard-of-care CT or MRI neuroimaging; (2) the acquisition of both T2W and FLAIR sequences during the portable MRI examination.
Data exclusions	A total of 15 exams were excluded from this analysis based on pre-established exclusion criteria. Nine exams were excluded due to the patient having a body habitus that prevented full brain insertion into the scanner's head coil and produced an incomplete field-of-view. Six exams were severely motion degraded and did not yield interpretable results. These 15 exams were excluded from all experiments included in the manuscript.
Replication	To verify the reproducibility of the experimental findings, we used a panel of blinded raters, all with established expertise reading clinical neuroimaging studies but with a range of experience. All raters were able to successfully adjudicate neuroimaging studies and provide an assessment. Each blinded rater independently assessed the 144 exams one time.
Randomization	This analysis is an observational study in which all subjects received a portable MRI examination
Blinding	Raters were blinded to all clinical and demographic data during data analysis. The investigators were not blinded to group allocation during data collection. Blinding investigators was not possible as they were required to screen the patients for study eligibility.

Reporting for specific materials, systems and methods

We require information from authors about some types of materials, experimental systems and methods used in many studies. Here, indicate whether each material, system or method listed is relevant to your study. If you are not sure if a list item applies to your research, read the appropriate section before selecting a response.

Materials & experimental systems

n/a	Involved in the study	
☒	☐	Antibodies
☒	☐	Eukaryotic cell lines
☒	☐	Palaeontology and archaeology
☒	☐	Animals and other organisms
☐	☒	Human research participants
☒	☐	Clinical data
☒	☐	Dual use research of concern

Methods

n/a	Involved in the study	
☒	☐	ChIP-seq
☒	☐	Flow cytometry
☐	☒	MRI-based neuroimaging

Human research participants

Policy information about studies involving human research participants

Population characteristics	Human research participants included in this analysis as patient participants had a mean age of 64±14 years. The cohort was comprised of 81 males and 51 females who were diagnosed with intracerebral hemorrhage (n=50), ischemic stroke (n=44), or no intracranial abnormality (n=38). Race and baseline medical history were only collected for subjects presenting with intracerebral hemorrhage and ischemic stroke. Subjects identified as white (n=64), black or African American (n=13), asian (n=8), other (n=6), or unknown (n=3). Subjects reported a previous stroke (n=12), hypertension (n=58), hyperlipidemia (n=36), diabetes mellitus (n=21), and/or atrial fibrillation (n=14). Non-patient healthy controls had a mean age of 41±11 years and included 28 males and 10 females.
Recruitment	From July 2018 to March 2020, portable MRI examinations were performed under a research protocol and participants provided informed consent. From March 2020 to November 2020, the device operated under FDA clearance and subjects received portable MRI exams as part of clinical care. To avoid disruption to the acute clinical workup of patients presenting with stroke, subjects were approached for recruitment a few days after hospital admission on average. As a result, the majority of the portable MRI examinations largely occurred within the subacute phase, resulting in a potential self-selection bias on timing of pathology. Moreover, when the device operated as a research study, critically ill patients were not enrolled as frequently due to the severity of their illness and thus, patients in the first half of the study typically had lower NIHSS and discharge mRS values.<br><br>Non-patient research participants scanned at Hyperfine HQ were recruited by distribution of flyers to a large group. Interested volunteers then contacted Hyperfine contacts, traveled to Hyperfine HQ in Guildford, CT, and signed the informed consent form prior to participating.
Ethics oversight	All patient data collected at Yale New Haven Hospital was performed under an Institutional Review Board (IRB) protocol approved by Yale Human Research Protection Program. All healthy control imaging at Hyperfine HQ was performed under a protocol approved by the Western Institutional Review Board-Copernicus Group (WCG) local IRB .

Note that full information on the approval of the study protocol must also be provided in the manuscript.

Magnetic resonance imaging

Experimental design

Design type	Observational
Design specifications	All subjects received one 17:51 minute portable MRI brain examination. Subjects with prolonged ICU admissions and willingness to undergo serial neuroimaging received additional portable MRI examinations throughout their ICU stay.
Behavioral performance measures	N/A, this study used structural magnetic resonance imaging (not functional) and did not require participants to undergo any behavioral performance measures or tasks during examination.

Acquisition

Imaging type(s)	Structural
Field strength	0.064 T
Sequence & imaging parameters	For T2W fast spin echo (FSE) imaging, relevant acquisition parameters were organized as follows (RC8/RC5/RC3): echo time [TE] = 252.6/252.3/200.5 ms, repetition time [TR]= 2200/2000/2000 ms, echo train length=80/72/64, number of averages=1/1/1, resolution=1.5x1.5x5 mm3/1.5x1.5x5 mm3/1.7x1.7x5 mm3, slices=36/36/36, acquisition time: 7:01/5:28/8:39 min. For FLAIR FSE, relevant acquisition parameters were: [TE]= 227.5/172.6/155.28 ms, [TR] =4000/1000/1000 ms, echo train length=80/48/48, number of averages=1/1/1, resolution: 1.6x1.6x5 mm3/1.5x1.5x5 mm3/1.7x1.7x5 mm3, slices=36/36/36, acquisition time=9:29/8:11/8:35 min.
Area of acquisition	Whole brain

Diffusion MRI ☐ Used ☒ Not used

Preprocessing

Preprocessing software	No preprocessing software was used.
Normalization	No spatial normalization was applied.
Normalization template	No spatial normalization was applied.
Noise and artifact removal	The portable MRI scanner operates in normal ICU rooms without RF shielding. The scanner has hardware noise detection coils and software to separate MRI signal from electromagnetic magnetic interference from the surrounding environment.
Volume censoring	No volume censoring was applied.

Statistical modeling & inference

Model type and settings	Study did not include functional MRI.
Effect(s) tested	Study did not include functional MRI.

Specify type of analysis: [x] Whole brain [ ] ROI-based [ ] Both

Statistic type for inference (See Eklund et al. 2016)	Study did not include functional MRI.
Correction	Study did not include functional MRI.

Models & analysis

n/a	Involved in the study
[x]	[ ] Functional and/or effective connectivity
[x]	[ ] Graph analysis
[ ]	[x] Multivariate modeling or predictive analysis

Multivariate modeling and predictive analysis	Manual and ABC/2 hematoma volumes on pMRI sequences (T2W and FLAIR) were correlated with National Institutes of Health Stroke Scale (NIHSS) obtained at the closest time to pMRI examination and modified Rankin score (mRS) at discharge using unadjusted and adjusted linear regression models. Adjusted linear regression models included sex, race, and age.
